# Gas Sensors Based on Molecular Imprinting Technology

**DOI:** 10.3390/s17071567

**Published:** 2017-07-04

**Authors:** Yumin Zhang, Jin Zhang, Qingju Liu

**Affiliations:** 1 School of Physics and Astronomy, Yunnan University, 650091 Kunming, China; Zhang_Yumin88@163.com; 2 School of Materials Science and Engineering, Yunnan Key Laboratory for Micro/Nano Materials & Technology, Yunnan University, 650091 Kunming, China

**Keywords:** gas sensor, molecular imprinting technology, quasi-molecular imprinting technology

## Abstract

Molecular imprinting technology (MIT); often described as a method of designing a material to remember a target molecular structure (template); is a technique for the creation of molecularly imprinted polymers (MIPs) with custom-made binding sites complementary to the target molecules in shape; size and functional groups. MIT has been successfully applied to analyze; separate and detect macromolecular organic compounds. Furthermore; it has been increasingly applied in assays of biological macromolecules. Owing to its unique features of structure specificity; predictability; recognition and universal application; there has been exploration of the possible application of MIPs in the field of highly selective gas sensors. In this present study; we outline the recent advances in gas sensors based on MIT; classify and introduce the existing molecularly imprinted gas sensors; summarize their advantages and disadvantages; and analyze further research directions.

## 1. Introduction

### 1.1. Significance of Gas Sensors

In the past few decades, the air quality of the globe has been declining, and many people die each year from indoor air pollution. The second United Nations Environment Assembly pointed that there are many people dying each year from indoor air pollution. Usually, the indoor air pollutants are composed of a series of volatile organic compounds (VOCs), which are generally harmful to human body. Those with low molecular weights (less than 100 Da) are particularly toxic, such as acetone [1], benzene [2], methanol [3,4], formaldehyde [5,6] and so on. Therefore, an accurate way to detect the toxic air pollutants is needed urgently. 

The traditional detection methods include spectrophotometry [7], chromatography [8], electrochemical methods [9], catalytic luminescence methods [10] and gas sensors [11]. Among them, the spectrophotometric method has the advantages of fast detection and low cost. However, this method requires a professional spectrophotometer, cannot be commercialized and needs extensive application. At the same time, there are also disadvantages, such as low accuracy, strict preparation of reagents and being easily affected by other factors (such as temperature and time of detection). The chromatography method is precise and fast, but there are also problems. This method needs special equipment that is expensive, in addition to having a large volume and high cost. It is also hard to separate isomers of the target reagent using chromatography. The electrochemical methods possess good stability and sensitivity, but the electrochemical sensors are more expensive. Furthermore, it has a short lifespan, and the detection process is susceptible to interference. The catalytic luminescence method, developed in recent years, is a new method for detection of toxic gases. Although the detection is of high sensitivity and good selectivity, the wide application of this method is restricted due to the complex equipment required and its high cost. Gas sensor methods are used to detect toxic gases with a high sensitivity and simple operations. The gas sensors are small devices available at a low cost, being suitable for real time monitoring and useful for detection of indoor air pollutants. Among many types of gas sensors, oxide semiconductor gas sensors are the mainstream products. The semiconductor gas sensor has been widely favored for the past twenty years due to its high sensitivity, stable performance, low price, small size, ease of use and so on. However, despite all these advantages, the semiconductor gas sensor still has some drawbacks, such as non-ideal selectivity for certain gases. When molecular imprinting technology (MIT) is used in gas sensors, there is an improvement in selectivity.

### 1.2. Molecular Imprinting Technology in Gas Sensors

**Figure 1:**
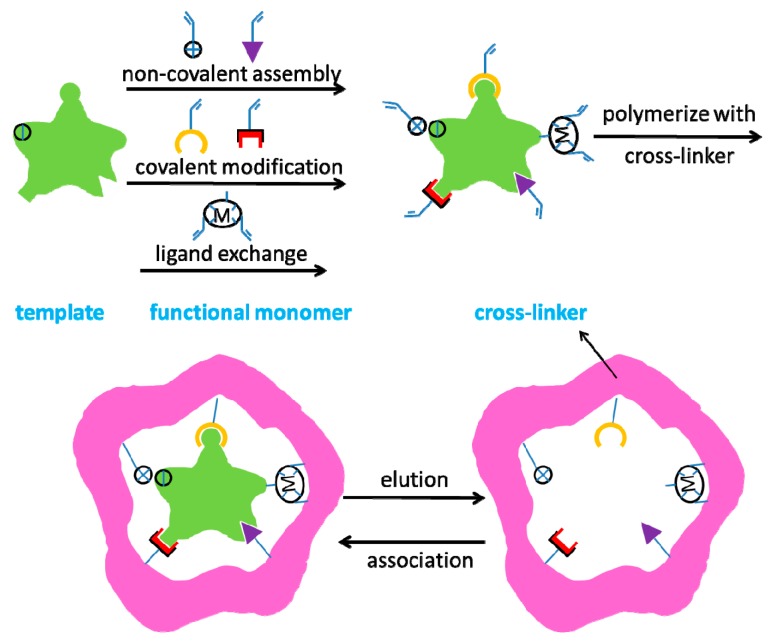
Schematic diagram of molecular imprinting process.

How can MIT improve selectivity? We should first learn about the features of MIT, which first appeared in 1930s. MIT provides a straightforward route for creating binding sites for a desired template, comparable to those of natural antibodies [12,13]. During the fabrication process, the template and functional monomers first form a self-assembled complex by covalent or noncovalent bonds where the functional monomers surround the template. Polymerization is then processed to support this self-organized configuration in place, followed by removal of the imprinted template from the polymeric networks, which thereby leaves behind binding sites complementary to the template. In this way, the match between the template and binding sites constitutes an induced molecular memory, which makes the prepared imprinted polymers capable of recognizing the templates as illustrated in Figure 1. In comparison with antibodies, molecularly imprinted polymers (MIPs) have the merits of easy preparation, reusability, and robustness for chemical and physical stresses. The molecularly imprinted gas sensors (MIGS) can be used under harsh conditions, including elevated temperature and pressure, presence of metal ions and organic solvent. Furthermore, MIGS can be fabricated using standard microchip fabrication protocols, such as photolithography, and have an extended lifetime. MIGS could benefit from the new development of nanosized-imprinted materials (“plastic antibodies”). The nanosized-imprinted materials can significantly improve binding kinetics, which represents a challenge in the development of sensors for real-time analysis. Furthermore, it would be a straightforward application in microfluidic devices. The essentials of MIT include functional monomers, interactions, initiator and elution [14,15]. As discussed above, molecular imprinting has the features of conformation reservation, recognition specificity, environmental tolerance and reusability, which have laid a solid foundation for the application of MIT in the fields of gas sensors [16].

### 1.3. Synthesis Strategies of Molecularly Imprinted Gas Sensors

The synthesis of MIGS can be divided into two categories: the molecular imprinting method, and the quasi-molecular imprinting method.

#### 1.3.1. Molecular Imprinting Method

The methods in this section contain all the reagents needed to be used in preparing the MIPs: template molecule, functional monomer, cross-linker, initiator and solvent. Functional monomers and a mass of cross-linkers are typically polymerized in the presence of the template molecules. Subsequently, the templates were removed from the polymeric matrix, which leaves behind some cavities. There are binding sites inside the cavities that are complementary to the template molecules in size, shape and functionality [17,18,19,20]. During these processes, the specific groups of functional monomers are oriented toward the desired binding sites within the cavities according to the structure, size and shape of the template [21]. In this manner, the preparation of MIPs is based on three steps: (1) the pre-interaction between the template molecule and the functional monomer(s); (2) the formation of a rigid polymeric matrix around the template molecule and the functional monomer(s) with a considerable number of cross-linkers; and (3) the removal of the template molecule [22,23].

Gas sensors in this category possess good stability and good selectivity. However, they have a long response and recovery time, due to the binding/elution process between the polymer matrix and the target gas molecules (the template molecules).

#### 1.3.2. Quasi-Molecular Imprinting Method

This sort of method is based on the MIT concept, although it does not strictly follow the process. Essentially, there is no exact functional monomer, initiator or cross-linker. The samples are prepared following normal ways, such as the sol–gel method or hydrothermal method. The samples are just synthesized or dried in a specific atmosphere formed with the target gases to make the sample recognize the target gases to enhance the selectivity.

## 2. Molecularly Imprinted Gas Sensors

### 2.1. Molecularly Imprinted Organic Gas Sensors

**Figure 2:**
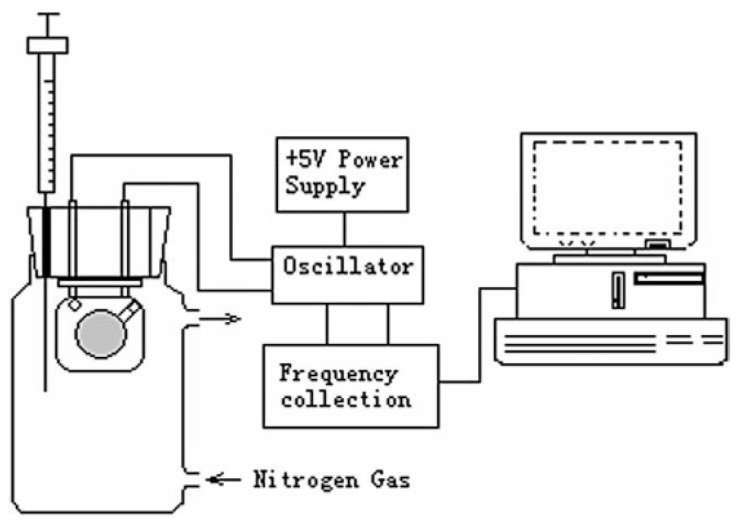
The schematic diagram of the sensor setup [24].

**Figure 3:**
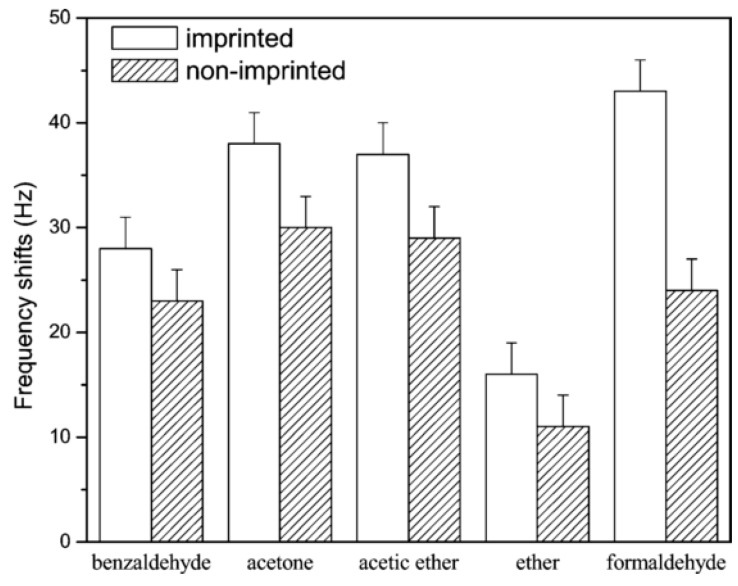
The resonance frequency response of the sensors to several analytes [24].

MIT has usually been used in the recognition or separation of biomacromolecules. Studies for recognition of toxic gases started in the early 21st century. MIT combined with quartz crystal microbalance (QCM) could potentially improve both the selectivity and sensitivity of gas sensors, especially the sensitivity. The limit of detection of QCM-based sensors can be less than other sensors fabricated with the same materials. Hu et al. [24] reported that a sensor fabricated by the piezoelectric method combined with MIT could selectively detect formaldehyde molecules. The idea was based on combining the tiny mass detection of QCM with the MIPs. Noncovalent MIP production was utilized in this experiment. Formaldehyde was used as a template, while methacrylic acid (MAA), ethylene glycol dimethacrylate (EGDMA), 2,2’-azobis(2,4-dimethyl)valeronitrile (AMVN) and toluene were used as the functional monomer, cross-linker, initiator and solvent, respectively. The schematic diagram of the sensor setup is shown in Figure 2. The sensor performance (sensitivity or selectivity) was characterized by resonance frequency. In terms of the interaction between molecularly imprinted binding sites and template, the selectivity of molecularly imprinted sample to formaldehyde was partially enhanced compared to the non-imprinted ones, as shown in Figure 3.

**Figure 4:**
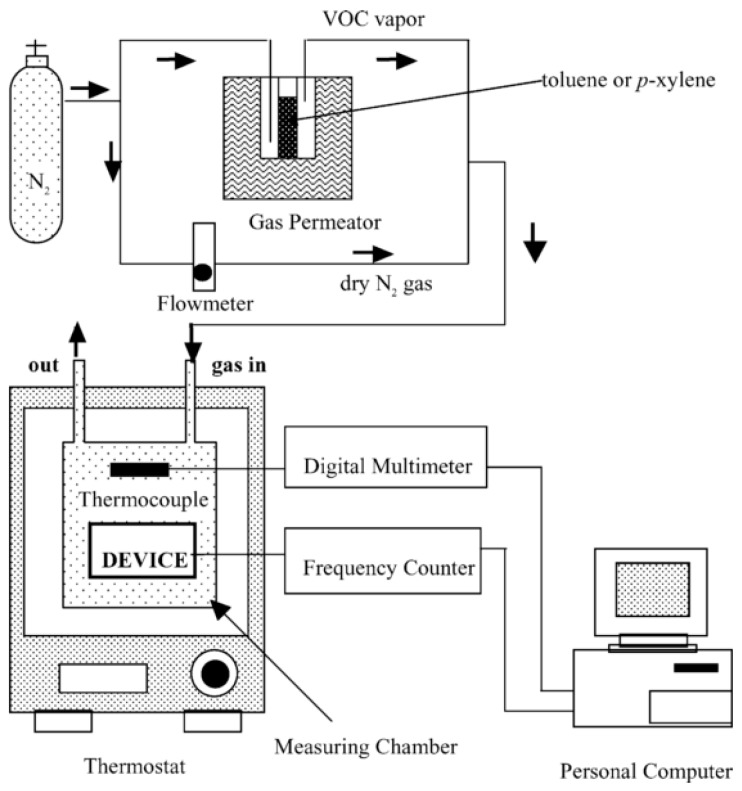
Experimental setup used to measure the volatile organic compounds sensor response [25].

**Figure 5:**
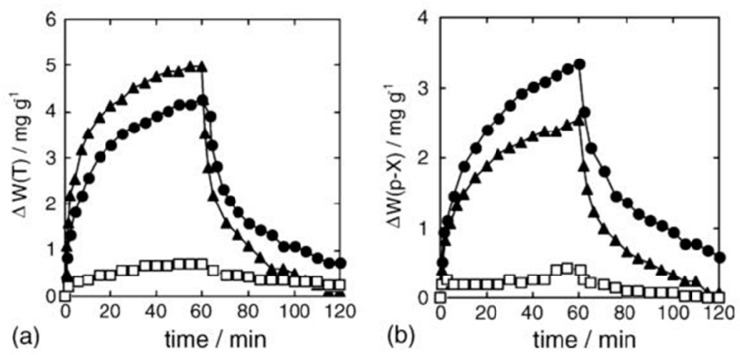
Sensor responses of MIP–PMMA and un-MIP–PMMA blend films measured using the QCM method at 30 °C: (**a**) toluene vapor sorption and (**b**) *p*-xylene vapor absorption (●: *p*-xylene-MIP-PMMA; ▲: toluene- MIP-PMMA; and ☐: un-MIP-PMMA) [25].

Matsuguchi et al. [25] used toluene and *p*-xylene as both template and solvent. This study used MAA, divinyl-benzene and benzoyl peroxide as the functional monomer, cross-linker and initiator, respectively, to fabricate a molecularly imprinted QCM-based toluene/*p*-xylene sensor. The gas measure setup is shown in Figure 4. The response to toluene or *p*-xylene and selectivity of the sensor was determined by the amount of absorbed toluene/*p*-xylene (△*W*(T), Figure 5). The results show that the device exhibits a good selectivity and high response to target gases, indicating that MIT could improve the selectivity of sensors to certain gases. 

Hawari et al. [26] demonstrated an e-nose sensor that could detect Limonene volatiles using MIP as the sensing material. In this research, Limonene, MAA, EGDMA, azodiisobutyronitrile (AIBN) and tetrahydrofuran (THF) were used as the template, functional monomer, cross-linker, initiator and solvent, respectively. The MIPs were prepared by spin-coating on an interdigitated electrode (IDE), with the properties of the sensor estimated by capacitance. The results showed that the sensor responds to Limonene volatile gases. Hawari et al. [27] also use the Interdigitated Electrode (IDE) structure to fabricate a sensor for detecting Alpha Pinene volatile gases by using MIP. Alpha Pinene, MAA, AIBN and EGDMA were respectively used as the template, functional monomer, initiator and cross-linker. The emission of Alpha Pinene was monitored by placing the IDE–MIP sensor into an actual ripe Harumanis mango. The results showed that the IDE–MIP sensor exhibited high sensitivity and selectivity responses towards alpha pinene when compared to non-imprinted polymers.

**Figure 6:**
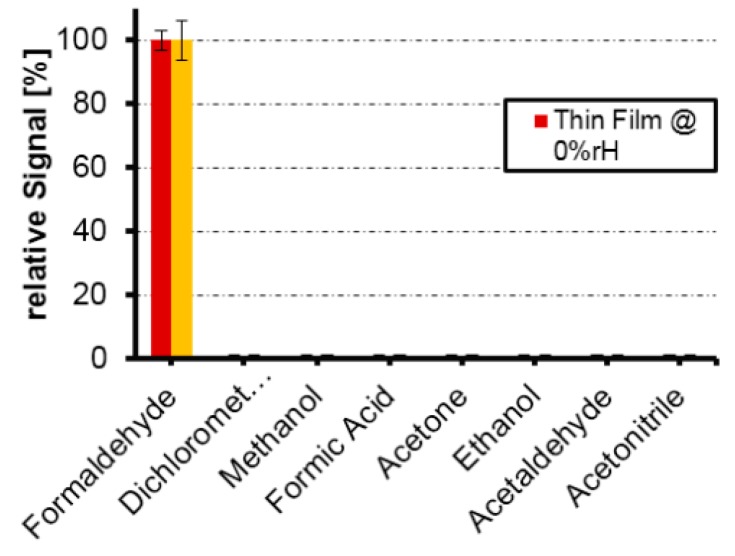
Selectivity patterns of MIP thin films and MIP nano-particles layers showing relative signals [30].

Ji et al. [28] fabricated a 2-methylisoborneol (MIB) QCM-based gas sensor using MIT. MIB was used as the template, MAA as functional monomer, EGDMA as cross-linker and AMVN as initiator and hexane as the solvent. The results showed that the QCM coated with a MIB imprinted polymer exhibit responses that were 1.1 ± 1.3 times higher than those of sensors coated with a non-imprinted polymer. The responses of the imprinted sensors to MIB were always the highest, and the detection limit was lowered to approximately 200 μg L^‒1^. Bunte et al. [29] prepared a MIP-coated QCM sensor to detect 2,4,6-trinitrotoluene (2,4,6-TNT) vapor. A PAA-MIP synthesized with chloroform showed best adsorption properties for TNT vapor. Direct measurements of the mass attachment with respect to the frequency decrease in the coated QCMs during vapor treatment showed a TNT-uptake of about 150 pg per μg MIP per hour. Lieberzeit et al. [30] examined a formaldehyde gas sensor using MIPs. A co-polymer thin film was prepared with styrene, MAA and EGDMA, before the thin film was coated with QCM to form a sensor. The sensor exhibits a detection limit of 500 ppb formaldehyde in dry air. These MIPs showed specific behaviors when tested against a range of VOCs, such as acetaldehyde, formic acid, dichloromethane and methanol, which can be seen in Figure 6. The device possesses great selectivity to formaldehyde.

**Figure 7:**
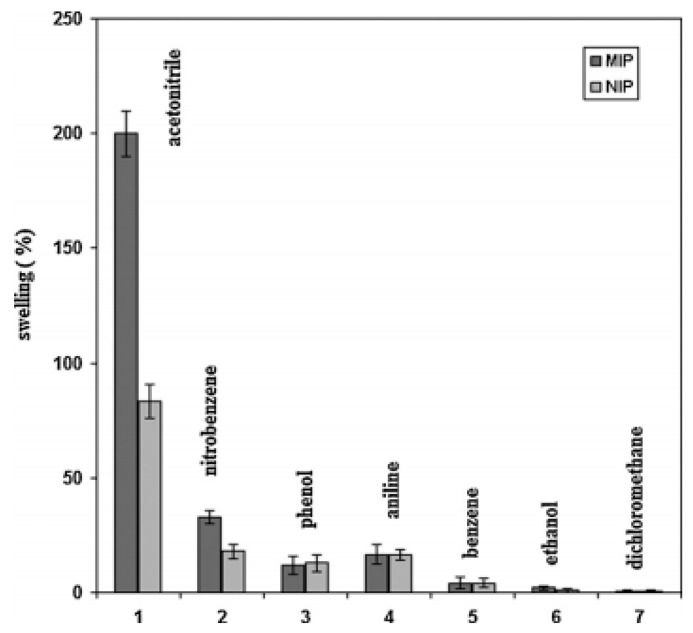
Comparison of the swelling intensities of the MIP and NIP in acetonitrile (1 mL) and other organic compounds (1 mM of them in acetonitrile, 10 mL) [31].

Alizadeh et al. [31] combined the imprinted polymeric particles with graphene to produce a nanocomposite chemo-resistor gas sensor. MAA was used as the first functional monomer, vinyl benzene as the second functional monomer, nitrobenzene as the template, divinylbenzene as cross-linker, and 2,20-azobisisobutyronitrile as radical initiator. The results showed that the sensor could recognize acetonitrile specifically, as shown in Figure 7. Wen et al. [32] presents a 300 MHz surface acoustic wave (SAW) gas sensor coated with MIPs, which was combined with a SAW oscillator. The MIP was prepared using o-phenylenediamine (o-PD) as a functional monomer and sarin acid as a molecular template, before being co-polymerized using cyclic voltammetry (CV). The sensitivity for the detection of dimethyl methyl phosphonate (DMMP) concentrations in a range of 1–100 mg/m^3^ was evaluated as approximately 96 Hz/mg/m^3^, with the threshold detection limit being up to 0.5 mg/m^3^. Walke et al. [33] reported a MIP to monitor explosive 2,4,6-TNT. Organically modified sol–gel polymer films with a thickness of submicrons were deposited on a waveguide surface as the sensing layer. Molecularly imprinted sol–gels were created for TNT using covalently bound template molecules linked to the matrix through 1 or 2 carbamate linkages. González-Vila et al. [34] focused on an optical fiber for formaldehyde gas detection. The optical fiber was coated with a layer of MIP based on polypyrrole, which is a conductive polymer. During the test, light is scattered when the target molecule attaches to the cavities present in the polymer. The sensor possesses good selectivity for formaldehyde. Jakoby et al. [35] applied a MIP thin film to a Love wave gas sensor. The sensor showed a certain response to 2-methoxy 3-methyl pyrazine (MMP). 

**Figure 8:**
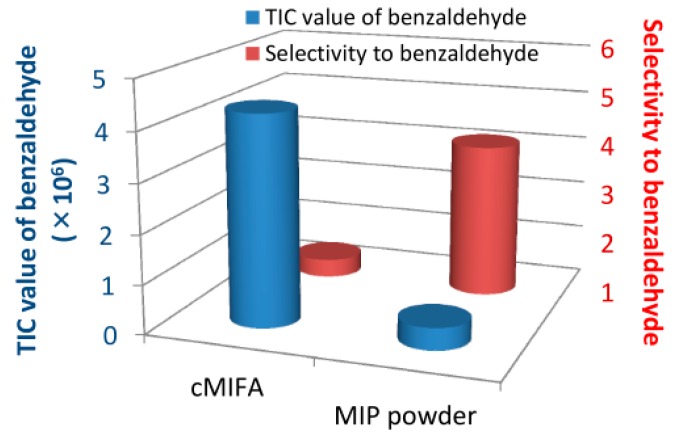
The comparison of the adsorption capability and selectivity of molecularly imprinted filtering adsorbents and MIP powder [39].

González-Vila et al. [36] fabricated a QCM gas sensor combined with sol–gel films for detection of parathion. Authors used silanes as functional monomers and parathion as the template to prepare MIPs. Following this, the MIPs were mixed with sol–gel films and then placed on a QCM. The molecularly imprinted QCM gas sensor possesses good selectivity for parathion, as the signal of the sensor to parathion is 3 times larger than that to other analytes. Jha et al. [37] designed a sensor array with volatile acids imprinted with 3-element QCM for the recognition of the odorous organic acids: propanoic acid, hexanoic acid and octanoic acid. Each element in QCM is in charge of detecting the odor of one acid and they all show good selectivity. Jha et al. [38] also developed a QCM sensor array to identify primary aldehydes in human body odor. Imahashi et al. [39] prepared a molecular imprinting filtering layer using benzaldehyde as template molecules. The molecular imprinting filtering layer was coated on a substrate to form a sensor. The sensor shows good selectivity to benzaldehyde, as shown in Figure 8.

Although some progress has been made in the early reports of MIGS, MIPs prepared in these reports usually use organic raw materials during the whole process and suffer from some drawbacks, such as poor electrical conductivity, moderate sensitivity and selectivity, and incomplete template removal. Therefore, the test process needs the help of QCM. Recently, new polymerization strategies have been proposed to deal with obtaining imprinted materials in order to improve the gas molecule detection of MIPs. Among these strategies, the sol–gel technique seems to be one of the simplest and most well-suited methods to obtain well-conducted MIPs.

### 2.2. Molecularly Imprinted Organic/Inorganic Hybrid Gas Sensors

In order to improve the conductivity of the MIPs, organic/inorganic hybrid MIPs have been examined lately using the sol–gel technique. Molecular imprinting-based sol–gel technology combines molecular imprinting and sol–gel techniques. The sol–gel technique is usually used to prepare solid-containing oxides or other compounds of inorganic or metal alkoxides. This process involves creating a solution, applying the sol–gel cure and heat treatment. The molecularly imprinted sol–gel technology is the method used to prepare a rigid material with an inorganic network structure, into which the template molecules are introduced via the sol–gel process. After the template molecules are removed, they show good affinity with the template molecules.

**Figure 9:**
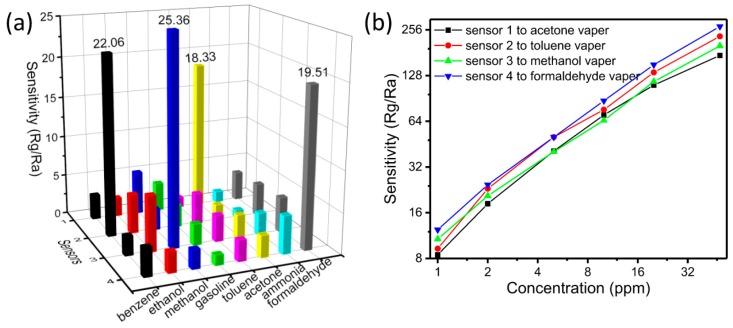
(**a**) The response of each sensor to eight types of analytes (2.5 ppm). (**b**) Response-concentration relationships to the target gases, Sensor 1 is the acetone sensor, sensor 2 is toluene sensor, sensor 3 is methanol sensor and sensor 4 is formaldehyde sensor.

**Figure 10:**
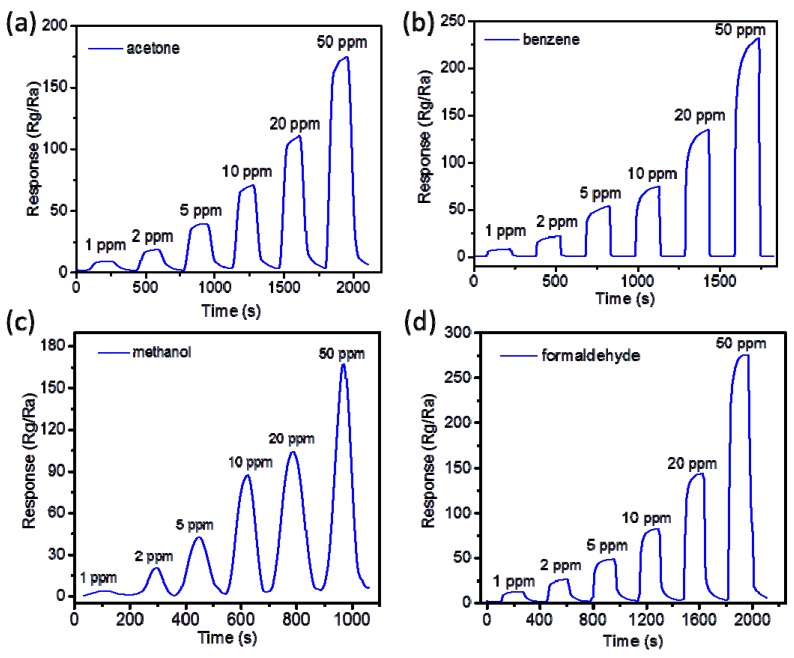
Rand recovery time of the: (**a**) molecular imprinted acetone sensor, (**b**) benzene sensor, (**c**) methanol sensor and (**d**) formaldehyde sensor.

Liu et al. [40,41] achieved a series of VOC sensors (formaldehyde, benzene, acetone, methanol and so on) based on Ag-doped LaFeO_3_ (ALFO) using the methods of the MIT combined sol–gel technique. In contrast to some single-metal oxide semiconductors, LaFeO_3_ (LFO) is a common perovskite-type oxide that exhibits p-type semiconducting behavior [42], and is a promising material with an abundance of functionalities, especially in the field of gas sensing. LFO possesses great potential for detecting pollutant gases because of its specific chemical and physical characteristics, including its large surface area, rich active oxygen lattice, good thermostability [43], controllable structure [44] and strong reducibility [45,46,47,48,49]. Thus, LFO is a more attractive gas-sensing material than other metal oxides. In addition, after LFO is combined with Ag, some Ag remains in the form of single atoms to act as catalyst mixtures in the matrix. Indeed, some of the Ag fills areas between the grains of the matrix, working to decrease the contact potential barrier and enhance the interfacial effects. This leads to a lower resistance and, thus, a lower operating temperature [50]. Therefore, ALFO is chosen to be used as the cross-linker (matrix) in Liu’s work. Most importantly, the selectivity of ALFO towards acetone, benzene, methanol and formaldehyde is successfully modulated by MIT. Using acetone as the template, N’N-methylene-bis-acrylamide (MBA) as the functional monomer and AIBN as the initiator, ALFO was prepared as an acetone gas-sensing material based on MIT. Similarly, using benzene, methanol and formaldehyde as the template, as well as choosing a proper functional monomer (formaldehyde for benzene, MAA for methanol and acrylamide for formaldehyde) and AIBN as the initiator, ALFO was then prepared as benzene, methanol and formaldehyde gas-sensing materials. Following this, these sensing materials were fabricated into heater-type gas sensors, based on which gas sensing properties were tested. The selectivity of each sensor is very good, which can be seen from Figure 9a. The relationship between response and concentration was also reported (Figure 9b). The increasing responses for the four sensors are closely linear to the concentration of each analyte, which indicates that the sensors can be used for continuous real-time monitoring of low concentration of VOCs. The response and recovery times of the molecular imprinted sensors to different concentrations of acetone, benzene, methanol and formaldehyde are shown in Figure 10. The response and recovery times were 44 s and 78 s (acetone); 63 s and 48 s (benzene); 40 s and 50 s (methanol); and 40 s and 54 s (formaldehyde). Nowadays, the MIT field has been dominated by the use of recognition and separation for organic macromolecules, such as proteins (molecular weight: 40,000–220,000 Da), enzymes (130,000–140,000 Da) and so on. However, for the small organic molecules, including VOCs (molecular weight is less than 100 Da), there are very few relevant reports. This is a great breakthrough in small organic molecule imprinting with the sol–gel technique based on a metal oxide semiconductor (ALFO). The simplicity and efficacy of this method has profound applications for the construction of applied gas sensors and has enormous potential.

Tang et al. [51] used electro-polymerization to synthesize the MIP layer on the TiO_2_ nano tube array/Ti sheet. The MIGS selectively detects formaldehyde in the ppm range at room temperature. Its sensitivity to formaldehyde is higher than that of acetone, acetaldehyde, acetic acid and ethanol.

## 3. Quasi-Molecularly Imprinted Gas Sensors

Another category of MIGS is prepared with quasi-MIT. According to the features of MIT, Huang et al. [52] suggested that a similar mechanism, the quasi-molecular-imprinting method, can be used in fabrication of gas sensors. Huang et al. put the target molecules in the preparation process in order to make the prepared materials recognize the target molecules to enhance the selectivity. They successively produced a CO sensor [52], ethanol sensor [53] and acetone sensor [54] using quasi-MIT. 

**Figure 11:**
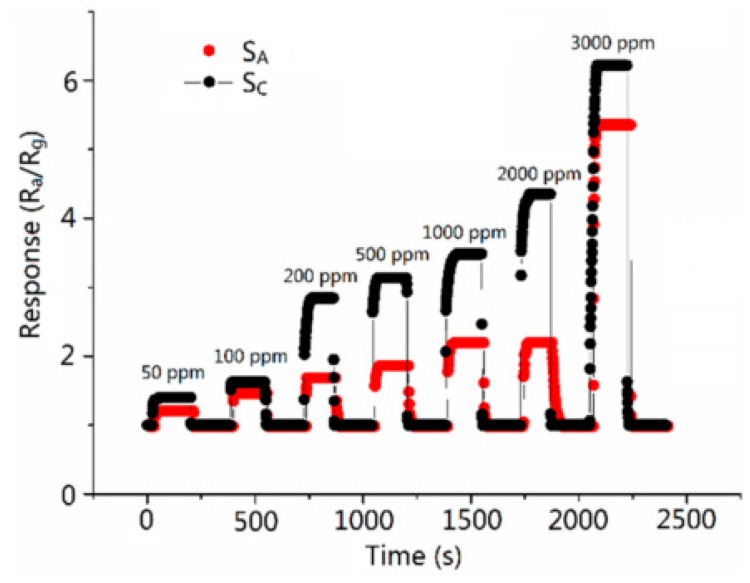
Responses to 50–3000 ppm of CO gas of imprinted (S_C_) and nonimprinted (S_A_) sensors at 300 °C [52].

To prepare the CO sensor, the authors first prepared SnO_2_ mesoporous nanomaterial using the hydrothermal method [52]. Following this, the prepared SnO_2_ film sensors were divided into two groups. One of the groups was dried in a carbon monoxide (which is the target gas) atmosphere to imprint CO on the surface of the material, while another was dried in the air for comparison. During the gas response test, the two groups of SnO_2_ sensors were exposed to CO gas with various concentrations ranging from 50 to 3000 ppm. The results shown in Figure 11 demonstrate that the imprinted sensor shows a faster response and recovery as well as a higher response compared to non-imprinted ones in all CO concentrations. It illustrates that quasi-MIT can enhance gas sensor performance. 

**Figure 12:**
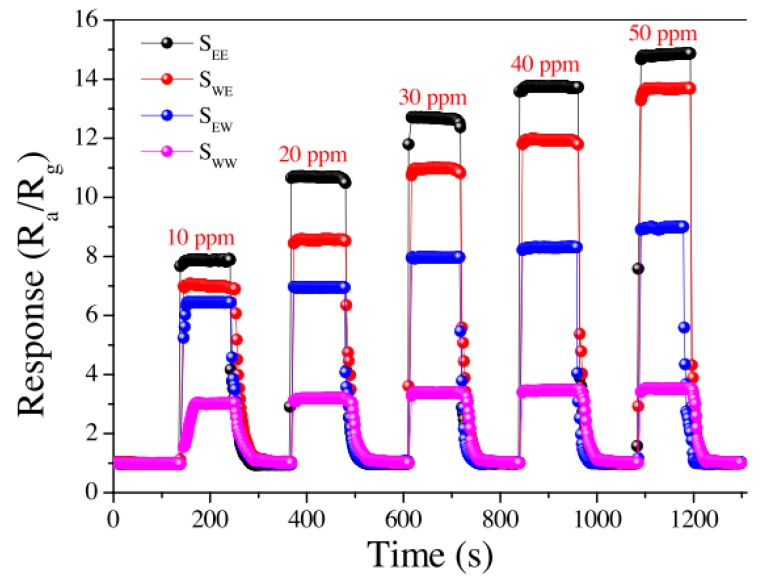
Responses to 10, 20, 30, 40 and 50 ppm of ethanol gas of imprinted and nonimprinted sensors at 300 °C [53].

For the ethanol sensor [53], the authors designed ethanol gas sensors based on SnO_2_ using the quasi-molecular-cluster imprinting mechanism. The target molecules (ethanol) were introduced during the SnO_2_ synthesis and device fabrication to acquire the specific structure that was more suitable for the adsorption and desorption of target molecules (ethanol gas). Under the same experimental conditions, nonimprinted sensors were prepared with deionized water. The synthesized SnO_2_ sensors coated by the imprinted and nonimprinted films were both exposed to ethanol gas, with concentrations ranging from 4 ppm to 100 ppm. The imprinted sensor exhibited the best and fastest response and recovery (Figure 12). 

**Figure 13:**
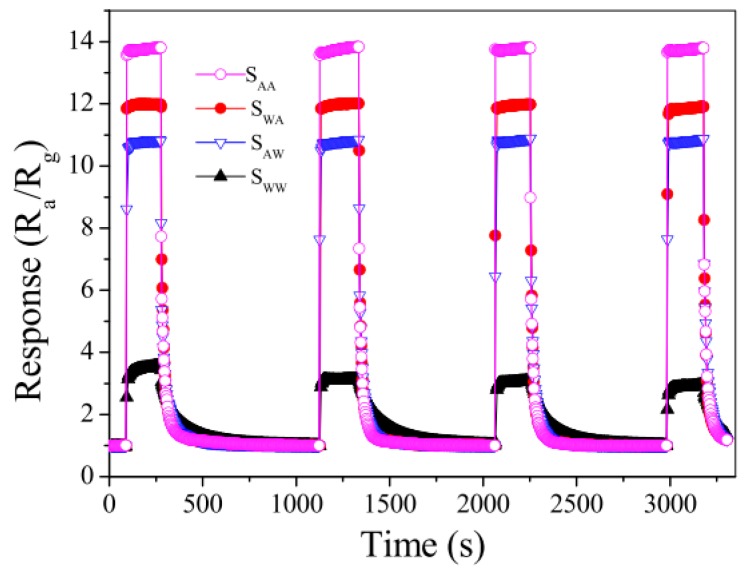
Responses to 50 ppm acetone gas for S_AA_, S_WA_, S_AW_ and S_WW_ at 250 °C [54].

For the acetone sensor [54], the method was very similar to that of the ethanol sensor mentioned above. Acetone solutions were introduced during the synthesis of SnO_2_ nanomaterials or/and device fabrication to produce appropriate structures more suitable for the adsorption and desorption of acetone gas. Similarly, there were imprinted and nonimprinted sensors. The synthesized sensors were exposed to acetone gas with various concentrations from 50 ppb to 100 ppm. The sensor produced by incorporating acetone both during the nanomaterial synthesis and device fabrication exhibits the best performance in terms of sensitivity, response recovery and reproducibility (Figure 13). 

The quasi-MIT concept is novel, and it is easy to implement. In this way, even inorganic gas sensors could be achieved. Although the mechanism is not very clear, and the author's speculations have not been completely confirmed, quasi-MIT provides a new idea in the molecular imprinted gas sensors, especially those for inorganic gases.

## 4. Conclusions

The molecular imprinting technique has good application prospects in the field of gas sensors, as molecular imprinted nanomaterials are suitable in sensing devices. For better performance, there are several problems that need to be resolved, such as the lack of a general synthesizing protocol, the non-uniformity of binding sites, the inorganic gas molecule recognition and improper adherence with the sensor surface. Exploring composites and hybrids of organic/inorganic polymers for developing imprinted materials in gas sensing would be of great value. An estimated trend in the gas sensors is toward miniaturization and sensor arrays. For example, certain sensors based on detection of VOCs integrated in one sensor array with different sensing units could be applied to simultaneous detection of multiple VOCs. In addition, materials, such as LaFeO_3_ or SnO_2_, can be fabricated as gas sensors to respond to different gas molecules with MIT and the (quasi-)molecular imprinting technique. In summary, any target molecules could be recognized by molecularly imprinted gas sensors as needed. In addition, by integrating different molecular imprinting gas sensors in an array, multiple kinds of gases could be detected simultaneously. This effectively expands the applications of molecular imprinting techniques in the field of gas sensors.
